# Understanding the Extent of Adolescents’ Willingness to Engage With Food and Beverage Companies’ Instagram Accounts: Experimental Survey Study

**DOI:** 10.2196/20336

**Published:** 2020-10-27

**Authors:** Samina Lutfeali, Tisheya Ward, Tenay Greene, Josh Arshonsky, Azizi Seixas, Madeline Dalton, Marie A Bragg

**Affiliations:** 1 Stanford Graduate School of Business Palo Alto, CA United States; 2 Department of Population Health NYU Grossman School of Medicine New York, NY United States; 3 Dartmouth Geisel School of Medicine Lebanon, NH United States; 4 School of Global Public Health New York University New York, NY United States

**Keywords:** social media, Instagram, social media marketing, food industry, adolescents, adolescent health

## Abstract

**Background:**

Social media platforms have created a new advertising frontier, yet little is known about the extent to which this interactive form of advertising shapes adolescents’ online relationships with unhealthy food brands.

**Objective:**

We aimed to understand the extent to which adolescents’ preferences for Instagram food ads are shaped by the presence of comments and varying numbers of “likes.” We hypothesized that adolescents would show the highest preferences for ads with more “likes” and comments. We predicted that these differences would be greater among adolescents who were “heavy social media users” (ie, >3 hours daily) vs “light social media users” (ie, <3 hours daily).

**Methods:**

We recruited Black and non-Latinx White adolescents (aged 13-17 years; N=832) from Dynata, a firm that maintains online participant panels. Participants completed an online survey in which they were randomized to view and rate Instagram food ads that either did or did not show comments. Within each condition, adolescents were randomized to view 4 images that had high (>10,000), medium (1000-10,000), or low (<100) numbers of “likes.” Adolescents reported ad preferences and willingness to engage with the brand.

**Results:**

Adolescents rated ads with medium or high numbers of “likes” higher than ads with few “likes” (*P*=.001 and *P*=.002, respectively). Heavy social media users (>3 hours/day) were 6.366 times more willing to comment on ads compared to light users (*P*<.001).

**Conclusions:**

Adolescents interact with brands in ways that mimic interactions with friends on social media, which is concerning when brands promote unhealthy products. Adolescents also preferred ads with many “likes,” demonstrating the power of social norms in shaping behavior. As proposed in 2019, the Children’s Online Privacy and Protection Act should expand online advertising restrictions to include adolescents aged 12 to 16 years.

## Introduction

Globally, young people are spending almost 3 hours per day on social media on average [1]. In the United States, more than 73% of adolescents report using social media daily, 27% of whom report logging in to at least one platform (eg, Snapchat, Instagram) each hour of their day [2]. Similar proportions of adolescents report using social networking sites daily in the United Kingdom, Australia, India, and Spain [3-6]. Food and beverage companies recognize the power of social media to reach young consumers and have greatly expanded their online presence. For example, one report showed that the digital media spending of McDonald’s—which is currently 36.1% of its ad budget—is expected to grow to 43.8% by 2023 [7]. Food companies also disproportionately market their products to youth of color, who already experience disproportionate rates of overweight and obesity [8]. Such targeting is concerning given the findings from numerous lab studies that youth who are exposed to food ads consume more food than youth who are exposed to nonfood ads [9]. In response to evidence on the link between exposure to food marketing and poor diet [10], 16 countries have now enacted policies that limit child-targeted food advertising, but only 5 of those countries include protections for adolescents aged 13 to 17 years, and only 3 address digital media specifically [11]. In the United States, two bipartisan senators proposed updating the Children’s Online Privacy and Protection Act (COPPA)—the US federal law designed to limit the marketing and collection of online data from children under the age of 13—in 2019 [12] and 2020 [13]. The proposed updates would eliminate racially targeted marketing practices, further limit companies’ ability to collect data from children, and expand COPPA protections to include adolescents younger than 16 years [12,13]. Yet, no studies have experimentally examined which social media features cause adolescents to interact with food companies on social media. Knowing which features trigger adolescents’ engagement with ads is critical for designing policies to reduce their exposure to ads.

Social norms theory informs why adolescents may be particularly susceptible to food ads on social media [14,15]. Adolescents are highly sensitive to peer behaviors [16-18], reward sensations [19], popularity cues [20], and pressure to conform [18,21]. Neurologically, the nucleus accumbens—the reward hub of the brain—of adolescents is more sensitive to reward compared to that of adults and children [19]. Common features of social media (eg, “like” button) and its highly interactive design may capitalize on these features of adolescent development. One neuroimaging study showed heightened activity in the nucleus accumbens among adolescents who viewed their own posts with high numbers of “likes” vs low numbers of “likes” [22]. In addition, esthetic differences between posts generated by friends compared to those generated by companies are subtle, making these ads a stealth threat.

Few studies have examined how adolescents engage with social media food ads, leaving major gaps in our understanding of how adolescents relate to brands on social media. One study found that about 6.2 million adolescents followed 27 food and beverage accounts on Twitter and Instagram [23], and qualitative analyses of 2000 social media food ads revealed that food ads featuring adolescents were 2.38 times more likely to contain interactive features (eg, encouraging viewers to “like” ads) compared to posts that featured adults [24]. Other cross-sectional studies have found that 70% of adolescents in a national sample “liked,” shared, or followed food and beverage brands on social media [25], or voluntarily uploaded images that contained a brand name or referenced a marketing campaign (eg, “Share a Coke”) [26]. Knowing which ad features cause adolescents to engage with food and beverage brands is critical for designing policies that shield adolescents from unhealthy ads.

The aim of the present study was to address these gaps by: (1) examining the extent to which adolescents’ willingness to “like” and engage with ads depends on whether the ad features low (<100), medium (1000-10,000), or high (>10,000) numbers of “likes”; (2) determining how the presence of positive comments vs no comments influences adolescents’ preferences and willingness to engage with the ad; and (3) comparing whether willingness to “like” or engage with the ads differs among adolescents who report heavy (>3 hours daily) vs light (<3 hours daily) social media use. We hypothesized that adolescents would show the highest preferences for ads with many vs few “likes” and comments, especially among heavy social media users.

## Methods

### Study Population

We recruited 1044 adolescents aged 13-17 years who identified as either Black/African American or non-Latinx White to complete an online survey. We recruited adolescents through Dynata, a firm that maintains online participant panels and recruits individuals from other websites, banner ads, and social media networks. Dynata uses a three-stage randomization process to recruit for surveys. To reduce selection bias, they invite interested panelists to “take a survey,” and no additional details are provided to the participants. Participants complete a proprietary quality control survey and are randomly assigned to surveys for which they likely qualify. The firm offers incentives (eg, cash, lottery, donations to charity) in exchange for participating in surveys. In the case of this study, parents were sent a link for this study and a consent form if their adolescent likely met eligibility criteria. Parents then provided consent before receiving a link to the assent form and then the survey was sent to the adolescents.

Of the 1044 adolescents who started the survey, 976 completed it and 884 correctly answered our data integrity question (ie, “Type Facebook in the box below.”) Fifty-two adolescents identified as belonging to a race/ethnicity other than Black/African American or non-Latinx White, and were excluded from the analyses. Our final sample included 832 adolescents. Table 1 presents the adolescents’ self-reported demographic characteristics and social media usage. Secondary analyses of comparisons by race/ethnicity are in preparation as a separate study.

**Table 1:** Demographic characteristics and social media usage of participants that completed the survey from January to June 2018.

Demographics			Total sample (N=832)		Comment Condition^a^ (n=414)		No Comment Condition^b^ (n=418)	
Age (years), mean (SD)			14.73 (1.67)		14.73 (1.68)		14.72 (1.66)	
**Sex, n (%)**								
	Male			426 (51.2)		209 (50.5)		217 (51.9)
	Female			406 (48.8)		205 (49.5)		201 (48.1)
**Race, n (%)**								
	Non-Latinx White			445 (53.5)		220 (53.1)		225 (53.8)
	Black/African American			387 (46.6)		194 (46.9)		193 (46.2)
**When do you use social media? n (%)**								
	Right when you wake up			305 (36.7)		158 (38.2)		147 (35.2)
	Before school			407 (48.9)		198 (47.8)		209 (50.0)
	On the way to school			309 (37.2)		138 (33.3)		171 (40.9)
	At school			258 (31.0)		133 (32.1)		125 (29.9)
	During lunch			381 (45.8)		179 (43.2)		202 (48.3)
	On the way home from school			323 (38.8)		147 (35.5)		176 (42.1)
	After school			381 (67.8)		290 (70.1)		274 (65.6)
	While doing homework			264 (31.7)		128 (30.9)		136 (32.5)
	After doing homework			390 (46.9)		205 (49.5)		185 (44.3)
	During dinner			158 (19.0)		87 (21.0)		71 (17.0)
	Before bed			497 (59.7)		243 (58.7)		254 (60.8)
	Right before going to sleep			234 (28.1)		114 (27.5)		120 (28.7)
**Do you have…? [check all that apply] n (%)**								
	Instagram			582 (70.0)		289 (69.1)		293 (70.8)
	Facebook			710 (85.3)		355 (85.8)		355 (84.9)
	Snapchat			410 (49.3)		201 (48.1)		209 (50.5)
	Tumblr			71 (8.5)		41 (9.9)		30 (7.2)
	Twitter			394 (47.4)		205 (49.5)		189 (45.2)
Average number of social accounts per participant based on responses to question above, mean (SD)			2.60 (1.20)		2.66 (1.17)		2.55 (1.19)	
Have you ever made a purchase through social media? n (%)			167 (20.1)		87 (21.3)		80 (19.5)	

^a^Comment Condition included ads that were Photoshopped with comments from Instagram users. We included comments that were positive in nature (eg, “I love [brand name]! Please sponsor me! [heart emoji]”).

^b^No Comment Condition included ads with the comment panel left blank, to appear as though Instagram users had not commented on the ad.

### Survey Procedures

Before data collection, we first tested the survey by having the research team take the survey and give feedback on its usability. Subsequently, Dynata representatives responsible for fielding our survey to their panelists tested the survey compatibility with their internal systems to ensure that participants could not take the survey more than once. They also tracked participants internally based on assigned user IDs to ensure that the participants would receive incentives. Parents provided informed consent, and adolescents provided assent after being given investigator contact details, being told that no identifying information would be collected and the survey would take about 15 minutes to complete, and that the study would involve viewing and rating images of consumer products. Adolescents then completed the online survey with a median completion time of 19 minutes. The online survey was hosted on Qualtrics, and Dynata required adolescents to complete the survey on a computer because the small screens on phones or tablets make it difficult to view the ads. Data were collected and analyzed in 2018. The New York University School of Medicine Institutional Review Board approved the study.

### Grouping

To select potential brands to include as stimuli, we searched the Instagram accounts of the fast food, beverage, and snack accounts with the most followers [24]. We selected McDonald’s, Pepsi, Coca-Cola, and Oreo because they have some of the highest numbers of adolescent followers among food and beverage brands [23], and because fast food, sugary beverages, and snacks are the categories of foods that are most heavily targeted to adolescents [27-30]. We used a random number generator to identify 4 numbers between 1 and 50, and used that number to randomly select an Instagram post from the official account for McDonald’s, Pepsi, Coca-Cola, and Oreo. For example, if the number was 8, the researchers selected the eighth most recent post on McDonald’s official Instagram account.

To create the “comment condition,” we used Photoshop to add comments from Instagram users onto the 4 ads we selected in the previous step. We included comments that were positive in nature (eg, “I love [brand name]! Please sponsor me! [heart emoji]”) to assess whether the presence of comments would encourage adolescents to “like” and comment on the ad. To create the “no comment condition,” we created the same comment panel but left it blank to appear as though Instagram users had not commented on the ad. Multimedia Appendix 1 shows an example of adding comments to an Instagram ad.

Within the “comment condition” and the “no comment condition,” we added high (>10,000), medium (1000-10,000), or low (<100) numbers of “likes” from social media users to each image. We based these cutoffs on data from a previous study showing that food and beverage company posts generate these ranges of numbers of “likes” [24]. This created a total of 6 groups to which participants were randomized: (1) comments + high “likes”; (2) comments + medium “likes”; (3) comments + low “likes”; (4) no comments + high “likes”; (5) no comments + medium “likes”; and (6) no comments + low “likes.” Qualtrics survey software provides capabilities to randomize participants automatically. We chose to use the automated randomization process to assign participants to the “comment” or “no comment” condition in a parallel randomization design. We were blinded to this process as it was automatic through Qualtrics. Although participants were not informed of the condition to which they were assigned, they could see the number of “likes” and whether posts had comments.

### Rating of Ads With Varying Numbers of “Likes” and Comments

Participants viewed an ad in their assigned group, and then answered the following questions: “How much do you like this ad?” (scale: 0-100 where 0 is not at all and 100 is very much); “Would you ‘like’ this ad on social media?” (yes/no); and “Would you comment on this ad on social media?” (yes/no). We used a continuous scale of 0-100 because previous studies have shown that a continuous rating scale is less subject to confounding variables compared to Likert scales, can capture more nuanced responses; in particular, one limitation of Likert scales is that the participants’ perceived difference between a “1—hate the product” and “2—somewhat dislike the product” is larger than the participants’ perceived difference between “2—somewhat dislike the product” and “3—neutral” [31,32]. We presented each ad in each condition in a random order to avoid bias, presenting only one ad and its corresponding questions on a page at a time.

### Self-Reported Social Media Use

Finally, participants completed a demographic survey and answered questions about their social media usage (Table 1). Participants saw 7 pages during the study, including the instructions page, the 4 ad pages, and the demographics and debriefing pages.

The primary outcomes were adolescents’ ratings of how much they liked the ad, the percentage of ads they were likely to “like,” and the percentage of ads on which they were likely to comment.

### Statistical Analysis

Analyses were conducted in SAS 9.4 software. We used multilevel regression for each outcome. Responses that were scored on a scale of 0-100 were analyzed using linear regression, whereas binary outcomes (yes/no) were analyzed using logistic regression. Because each adolescent rated multiple ads, each model included a random effect for the participant to account for the repeated measures.

Analyses that adjusted for time spent on social media used a median split at 3 hours, such that adolescents who reported spending more than 3 hours per day on social media were labeled “heavy social media users” and those who reported spending less than 3 hours per day on social media were labeled “light social media users.” Analyses were stratified according to whether or not adolescents were shown ads with comments and “likes” (comments condition) or just “likes” (no comments condition). Chi-square tests and *t* tests were conducted to determine if the randomization was successful and to verify that demographic characteristics did not differ between conditions. Because all of these tests were insignificant, demographic characteristics were not included in the models. The Holm-Bonferroni procedure was used to correct for multiple comparisons. We also compared responses to racially targeted food advertising among Black and White participants in a separate study that is currently under review elsewhere.

## Results

### Rating of Ads With Varying Numbers of “Likes” and Comments

The survey randomized 915 participants into two arms: 455 participants were allocated to the “comment condition” and 460 participants were allocated to the “no comment condition.” All participants who were assigned a condition completed that part of the survey. Since some participants incorrectly answered our data integrity question, we ultimately analyzed data from 414 participants in the “comment condition” and 418 participants in the “no comment condition.”

Across all ads in both conditions, adolescents reported liking the ads (mean 65.62, SD 27.06 on a 100-point scale) and 576 of 832 participants (69.2%) reported they would “like” one or more of the ads on social media. When rating ads in the “no comment condition,” ads with a higher number of “likes” received significantly higher ratings than ads with fewer “likes,” and were also associated with higher willingness by the adolescents to “like” the ads themselves (Table 2). In contrast, when rating ads in the “comment condition,” there were no significant differences in ratings or willingness to “like” the ads based on the number of “likes” the ads received.

**Table 2:** Ratings of ads with varying “likes” and comments.

Outcome Measures	Ads with <100 “Likes”		Ads with 1000-10,000 “Likes”			Ads with >10,000 “Likes”	
	No comment condition	Comment condition	No comment condition	Comment condition	No comment condition		Comment condition
How much do you like this ad? (0, not at all to 100, very much)	61.88 (1.34)	65.15 (1.25)	66.28 (1.34)^a^	65.77 (1.25)	67.74 (1.33)^a^		66.88 (1.24)
Would you “like” this ad on social media? (% who said “yes”)	65.7%	67.8%	67.3%	69.1%	70.7%^b^		74.6%
Would you comment on this ad on social media? (% who said “yes”)	37.9%	37.8%	34.2%	40.2%	37.1%		40.6%

^a^Holm-Bonferroni adjusted *P*<.001 comparing medium or high likes and low likes conditions.

^b^Holm-Bonferroni adjusted *P*<.02 comparing high likes and low likes conditions*.*

Across both conditions, 315 of 832 participants (37.9%) reported a willingness to comment on ads, and there were no significant differences in adolescents’ willingness to comment based on whether or not the ad had comments from other social media users or whether the ad had a high, medium, or low number of “likes.”

In the “no comments condition,” heavy social media users reported liking ads 8.614 points more than light users, controlling for the number of “likes” on the ad and the race of the participant (SE 2.263, *P*<.001). In the “comments condition,” heavy users reported liking ads 13.615 points more than light users, controlling for the number of “likes” on the ad and the race of the participant (SE 2.018, *P*<.001).

Further, when adjusting for the level of “likes” on the ad in the “comments condition,” heavy users were 6.366 times more willing to comment compared to light users (*P*<.001). When adjusting for the level of “likes” on the ad in the “no comments condition,” heavy users were 2.564 times more willing to comment compared to light users (*P*<.001).

There was no evidence of a differential response to the level of “likes” on an ad for any of the three outcome measures when comparing heavy and light users (ie, heavy users were not more responsive to the number of “likes” on an ad than light users).

### Self-Reported Social Media Use

Every adolescent in the sample reported having one or more social media account (Table 1). They reported spending on average of 4.81 hours (SD 4.66) per day on social media, although the median was 3.00 hours per day. Among the 832 participants, 167 (20.1%) reported having ever made a purchase through social media. On average, the adolescents had created their first social media account at 12.88 years old (SD 2.37).

## Discussion

### Principal Findings

This study demonstrated that adolescents preferred food ads on Instagram that featured many vs few “likes.” Adolescents were also more willing to engage with Instagram food ads (ie, through “likes”) when the ads featured many “likes” compared to few “likes.” Further, “heavy users” of social media preferred and were more willing to “like” ads compared to “light users,” who spend less than 3 hours per day on social media. This was also the first study to examine the interaction between the presence of comments and varying number of “likes.” Results showed that the presence of positive comments did not affect adolescents’ ad preferences, which suggests that “likes” may function as a more powerful social tool than comments. These data provide new insights into the effects of “likes” and comments on adolescents’ preferences for ads and willingness to engage with ads. These findings add to the literature on social media usage among adolescents by demonstrating that adolescents are highly willing to interact with ads. We also identified thresholds for the number of “likes” that may be required to shift adolescents’ willingness to “like” an ad. Specifically, adolescents were more likely to prefer and “like” ads with high (>10,000) vs low (<100) numbers of “likes.” However, there were no differences in their willingness to engage with ads featuring high “likes” (>10,000) vs medium “likes” (1000-10,000). These thresholds are concerning because companies, celebrities, and influencers who promote unhealthy products often have more followers than public health campaigns, and therefore may be able to generate more engagement with adolescents.

One particularly concerning set of findings is that heavy social media users reported higher preference for and willingness to engage with food and beverage ads compared to light social media users. Heavy social media users’ positive ad ratings may, however, simply result from familiarity with these types of ads. Although it is unclear how many social media ads adolescents see annually, one study found that 72% (n=101) of adolescents and children were exposed to one or more food ads during a 5-minute data collection period [33], and 44% of those ads promoted sugary beverages. Future research should capture more exposure data and identify the behavioral responses to advertisements between heavy and light users of social media. Although heavy social media use concerns parents and researchers alike [13,34], there are important positives to social media use [35], especially for marginalized groups seeking connectedness and acceptance [36-41].

Our findings reinforce other research on social media behaviors and the power of “likes.” The average age at which adolescents in our sample reported creating one or more social media account was 12.88 years, even though most social media sites aim to require users be 13 years of age. Similarly, one survey of 1786 parents of youth aged 8 to 18 years found that nearly half of the children started using social media at 12 years old [42]. In our ad rating questions, the adolescents reported higher preferences for and willingness to “like” ads that featured high numbers of “likes” compared to low numbers of “likes,” which is consistent with similar findings from other studies [43,44] and supports the possibility that social norms may drive “likes” among adolescents.

Additionally, our findings support the need for stronger protections in the COPPA in the United States, and international policies would also benefit from strengthening regulations regarding digital advertising. The findings suggest that adolescents may be highly susceptible to social media food ads, which is concerning given the link between exposure to food ads and poor diet [9]. Because it is possible for adolescents to follow social media accounts from other countries, the countries with enacted policies for food marketing should also address digital media. Further, food companies should expand the Children’s Food and Beverage Advertising Initiatives in the United States by enacting an international policy that would limit the promotion of unhealthy food and beverages posts that could be viewed by social media users in other countries.

Finally, 20.1% of the adolescents in our sample reported having purchased a product via social media. To our knowledge, these are the first data to document that adolescents report making purchases on social media. Although one design firm’s survey of 2000 Instagram users found 18% of users have made a purchase directly through Instagram, the sample did not include adolescents aged 17 years and younger [45]. Adolescents’ exposure to seemingly popular food ads (ie, those with many “likes”) may increase their willingness to purchase products on social media, which could be problematic if they purchase branded items (eg, clothing) that then increase brand loyalty and willingness to purchase, and subsequently consume, more unhealthy food and beverage products. These links between ad exposure and future purchases of unhealthy products are supported by studies that show how adolescents’ peers shape their online purchases [46], and that authentic and individualized social media advertising can enhance consumers’ relationships with brands and increase brand loyalty [47,48]. One industry report suggests that today’s adolescents display higher brand loyalty than previous generations, and that 66% of adolescents stated that once they find a brand they like, they will continue to buy that brand for a long time [49].

### Limitations

This study has several limitations. Our small sample (N=832) limits our ability to generalize findings on adolescents’ self-reported social media usage habits to other adolescents. It is also possible that the participants may have responded with social desirability bias to provide answers they thought the researchers might prefer. Survey responses, however, were anonymous, which reduces the possibility of social desirability bias. Although we found significant differences between heavy users and light users across all ad rating outcomes, additional research is needed to determine if these differences translate to increased susceptibility to advertising. Because we recruited participants from an online panel, our participants may use technology more or be more tech-savvy than the general population, which could impact our results. However, how often participants used social media varied in our sample. Finally, Instagram recently announced they will start hiding “likes” on Instagram users’ posts so that users can “focus on the photos and videos…not how many likes they get” [50]. Users will still be able to see the number of “likes” on their own posts, and it is possible that hiding “likes” will reduce adolescents’ engagement with ads because the ads will lack cues that indicate their popularity. Further, other social media sites such as Facebook and TikTok have not mentioned plans to remove “likes,” suggesting that the findings are still relevant to social media sites.

### Conclusions

This study demonstrates that adolescents preferred and were more willing to “like” Instagram food ads featuring many “likes” versus few “likes,” and “heavy social media users” preferred and were more willing to engage with food ads compared to “light social media users.” The findings on ad ratings demonstrate the power of “likes” in shaping adolescents’ behavior and preferences, especially considering that the near-constant use of social media is rising in this age group [2]. These results also support the possibility that Instagram’s recent decision to hide the “likes” feature may reduce adolescents’ willingness to “like” ads and therefore to reduce engagement with posts that may promote unhealthy habits. Nevertheless, policymakers should enact stronger protections in the COPPA to reduce adolescents’ exposure to unhealthy food and beverage ads that can shape poor diet habits and increase risks for developing diet-related diseases later in life [51]. Although interventions have been tested to improve media literacy and educate children and adolescents about deceptive marketing tactics, these interventions may not be effective in limiting marketing influence. Such interventions focusing on teaching critical viewing skills and skepticism of food advertising have resulted in only minor increases in self-reported skepticism of ads in children 8 years and older [52,53]. Further, research has shown that even when older children and teenagers are able to accurately recognize advertising, they are often unable to resist its influence when embedded in personalized content and trusted social media networks [54], underscoring the critical importance of policy solutions.

## Appendix

Multimedia Appendix 1 Example of adding comments to an Instagram ad.

## Abbreviations

COPPA: Children’s Online Privacy and Protection Act
