# Supplementary figures and images for: Understanding the Extent of Adolescents’ Willingness to Engage With Food and Beverage Companies’ Instagram Accounts: Experimental Survey Study
Source: JMIR Public Health Surveill. 2020 Oct 27;6(4):e20336. doi: 10.2196/20336 (PMC7655467; doi:10.2196/20336)

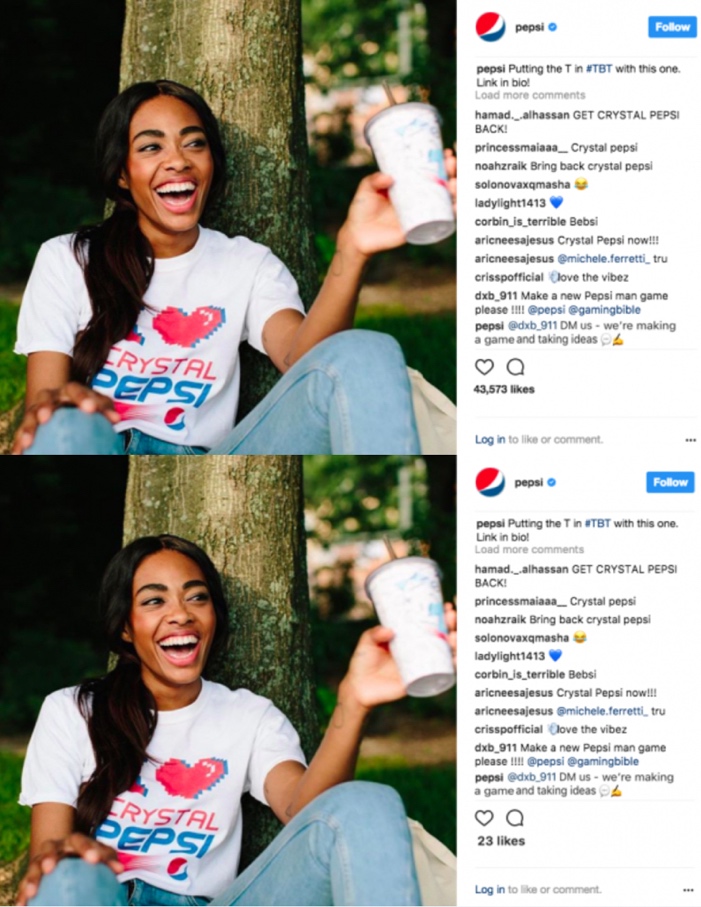

Supplement: Multimedia Appendix 1 [file publichealth_v6i4e20336_app1.docx]
